# Superficial Granulomatous Pyoderma of the Face: A Case Report and Review of the Literature

**Published:** 2012-12-10

**Authors:** Sarah M. Persing, Donald Laub

**Affiliations:** ^a^University of Vermont College of Medicine, Burlington; ^b^Fletcher Allen Health Care, Burlington, Vt.

## Abstract

**Introduction:** Superficial granulomatous pyoderma (SGP) is a rare variant of pyoderma gangrenosum (PG) and differs from classic PG in its indolent clinical course, lack of associated underlying disease, the finding of a granulomatous infiltrate on histology, and better prognosis with less-aggressive therapies. **Methods:** We report on a case of SGP involving the face following local reexcision of a biopsy scar. **Results:** The patient developed an indolent ulceration following an excision of an unfavorable scar on her face. **Discussion:** Superficial granulomatous pyoderma is usually responsive to conservative treatment with antibacterial or local anti-inflammatory agents. Facial involvement with SGP is particularly rare and tends to be more refractory to conservative management. Superficial granulomatous pyoderma involving the face appears to respond better to intensive treatment with systemic corticosteroids or immunosuppressants. To prevent poor cosmetic and functional outcomes in patients with nonhealing lesions, it is important for surgeons to recognize the clinical and histopathologic presentation of SGP and consider initiating a more aggressive management approach for SGP involving the face.

Superficial granulomatous pyoderma (SGP) is a rare, slowly progressive, and superficial variant of pyoderma gangrenosum (PG) that was first described by Wilson-Jones and Winkelmann in 1988.^1^ Both classic PG and SGP are progressive ulcerative diseases of the skin that may demonstrate pathergy, the phenomenon whereby ulcerative lesions can be induced at sites of skin trauma. Superficial granulomatous pyoderma differs from PG in its indolent clinical course, lack of associated underlying disease, the finding of a granulomatous infiltrate on histology, and better prognosis with less-aggressive therapies.^1^^,^^2^ To date, there are approximately 60 cases of SGP that have been described worldwide, with facial involvement present in only 6 of these cases.^1^^-^9 In this report, we describe a case of SGP involving the face and present a review of the literature.

## METHODS

J.M. is a 37-year-old woman who initially sought an evaluation of a depressed circular scar on her right cheek resulting from a shave biopsy 3 months previously. The biopsy results reportedly demonstrated benign pathology. The wound broke down after suture removal 9 days later and healed slowly with a scar that was larger than the original lesion. The patient had no prior problems with wound healing. She underwent local reexcision and closure of the biopsy scar. Tissue biopsy demonstrated granulomatous inflammation.

## RESULTS

The wound healed well until approximately 4 to 5 weeks after surgery when she noticed increasing erythema of her reexcisional scar. The wound began to dehisce and a culture grew methicillin-sensitive *Staphylococcus aureus.* Despite appropriate antibiotics, she had only slight improvement of her wound. A second biopsy once again demonstrated granulomatous inflammation, and mycobacterial and fungal cultures were taken on these tissue biopsies. No acid-fast bacilli were isolated; fungal culture grew *Candida parapsilosis*. She did not improve after a course of fluconazole, however. Additional laboratory workup showed negative or normal results.

**Figure 1:**
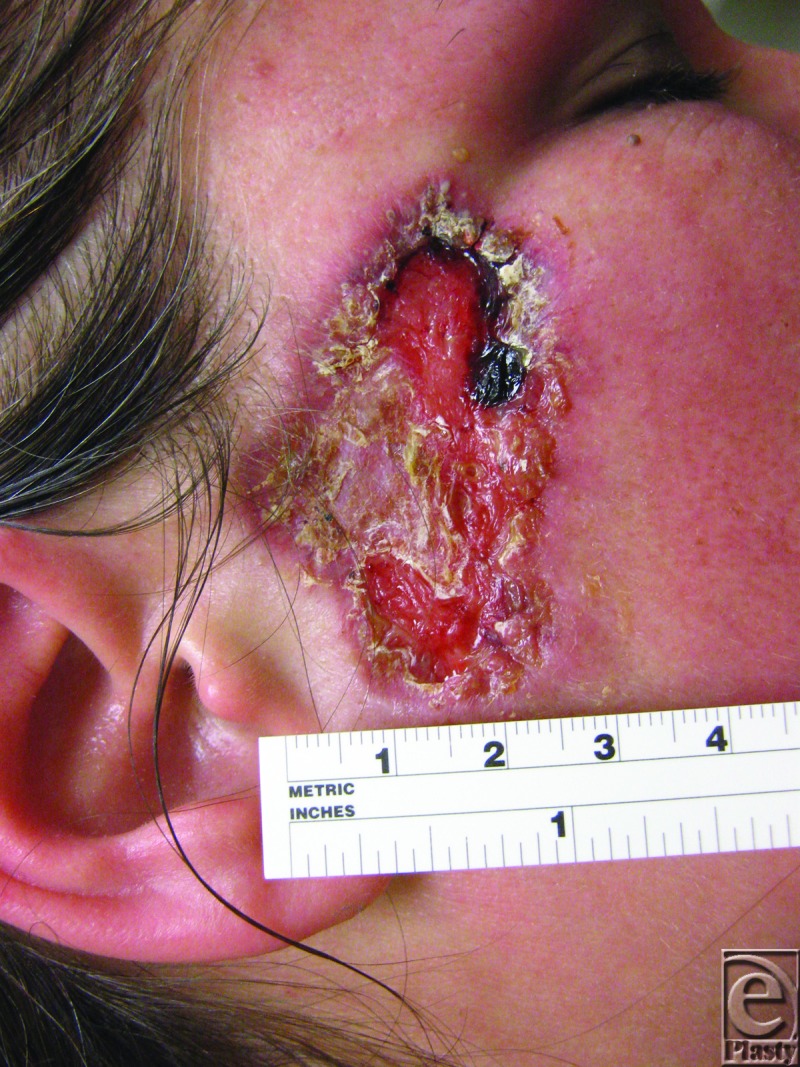
Ulcerated plaque at 5 weeks after reexcision of the biopsy scar.

**Figure 2:**
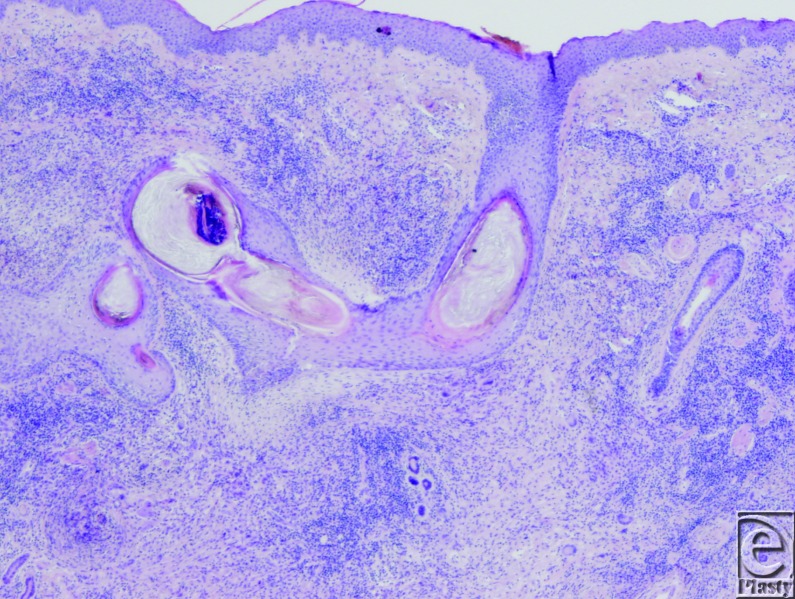
Tissue biopsy specimen showing reactive epidermal hyperplasia with neutrophil-rich inflammation and focal granuloma formation (hematoxylin-eosin, original magnification ×10).

Approximately 8 weeks after reexcision of the biopsy scar, she had a 5.2×3.2-cm ulcerated erythematous plaque with a pink rim on her right temple with granulation tissue at the bed (Fig 1). A third biopsy specimen was obtained from the reexcised lesion. This biopsy demonstrated reactive epidermal hyperplasia with neutrophil-rich inflammation, focal granuloma formation, and granulation tissue (Fig 2). There were no fungal, bacterial, or mycobacterial organisms identified with appropriate stains and culture.

**Figure 3:**
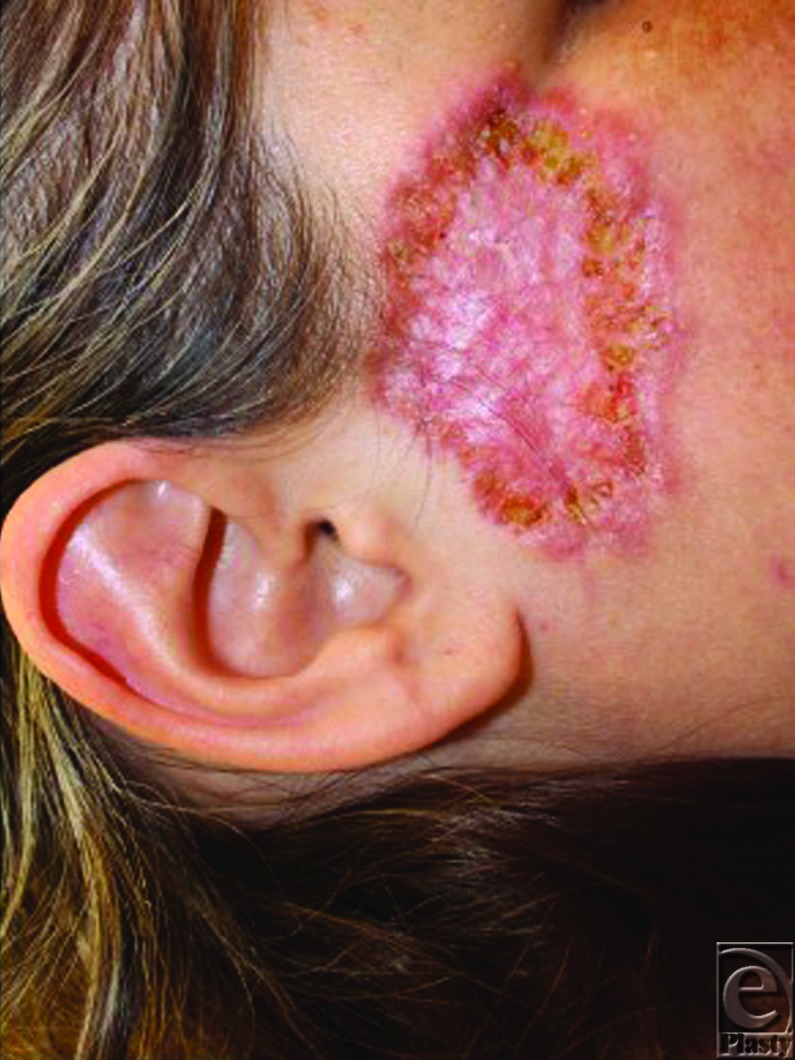
Healing lesion after 1 month of prednisone therapy.

The diagnosis of SGP was subsequently made on the basis of the clinical and histopathologic characteristics of her presentation. Treatment was initiated with oral prednisone 50 mg daily and mupirocin 2% ointment. At 1-month follow-up, substantial improvement was noted with significant reepithelialization from her prior evaluation (Fig 3).

## DISCUSSION

Superficial granulomatous pyoderma is a rare disease that is considered to be a variant of PG. The pathogenesis of SGP remains unknown; however, pathergy is believed to be the precipitating factor in most cases but not all. It has been suggested that SGP may be a localized delayed-type hypersensitivity reaction of the skin to a yet unidentified endogenous or exogenous organism or antigen.^1^^,^^2^^,^^9^

Superficial granulomatous pyoderma shares some similarities and is often mistaken for classic PG, but there are distinctive characteristics in the location, histologic features, prognosis, and treatment that are unique to SGP (Table 1). Clinically, the site of predilection for SGP lesions is the trunk. Superficial granulomatous pyoderma rarely affects the face. Unlike PG, SGP is not associated with serious underlying systemic, autoimmune, or other diseases.^10^ Superficial granulomatous pyoderma lesions tend to appear as a single, nontender, well-defined superficial ulcer with exophytic or vegetating clean granulations.^1^ Histopathologically, the ulcers show a characteristic 3-layered granuloma: an innermost zone of necrotic debris and neutrophils, a surrounding layer of granulomatous inflammation with histiocytes and giant cells, and an outer layer composed of plasma cells and eosinophils.^6^ The major differential diagnosis for SGP is classic PG. Other differentials include mycobacterial and fungal infections, foreign-body granuloma, vasculitis, halogenoderma, and other granulomatous diseases.

Effective treatment of SGP differs somewhat from PG. Systemic corticosteroids are first line in the treatment of PG but are usually not necessary to control SGP lesions.^8^ Superficial granulomatous pyoderma is generally responsive to conservative treatment with antibacterial or local anti-inflammatory agents. There are, however, some exceptions that require more aggressive therapies. Interestingly, in many of these intractable cases, SGP involved the face.^2^^,^^3^^,^^5^^,^^7^ It has been suggested that facial involvement with SGP may be more refractory to conservative treatments.^9^ These cases of SGP with facial involvement have been successfully treated with cyclosporine,^7^ intravenous immunoglobulin therapy,^5^ a combination of dapsone and oral corticosteroids,^2^ and more recently, infliximab.^3^ In all of these studies, conservative therapy was attempted but ultimately failed.

Pyoderma gangrenosum is believed to exist as a clinical spectrum with classic PG representing the more acute and debilitating end, while SGP represents a more indolent and benign opposite end.^6^ This concept may help explain why certain cases of SGP do not respond well to conservative treatment in that they may be exhibiting features that are more consistent with classic PG, in which case, milder therapies would be ineffective.

Superficial granulomatous pyoderma is a slowly progressive, relatively benign disease that generally has a favorable prognosis with conservative treatment and does not require systemic immunosuppressive agents. Facial involvement with SGP, however, may represent a more aggressive clinical entity that is distinct from typical SGP in nature and may require more intensive management at the onset. Delay in initiating appropriate treatment, combined with excessive trauma from multiple reexcisions, may result in poor cosmetic and functional outcomes in patients with SGP, especially for lesions involving the face.

For patient J.M. presented in this case of SGP, the appearance of the final scar is clearly suboptimal; however, it was judged that the risk of further pathergy was great with continued surgical management of this wound, so no attempt at scar revision was contemplated and appropriate medical management was initiated. We present this case to make plastic surgeons aware of a medical condition that may make plastic surgical intervention unpredictable and potentially more damaging. It is important for plastic surgeons to recognize the clinical and histopathologic presentation of SGP to avoid disease progression and permanent disfigurement.

## Figures and Tables

**Table 1:** Contrasting the differences between pyoderma gangrenosum and superficial granulomatous pyoderma

Feature	Pyoderma gangrenosum	Superficial granulomatous pyoderma
Location	Extremities	Trunk
Lesion	Liquefying ulcer	Granuloma
Border	Undermined	Vegetative
Base	Necrotic	Clean
Depth	Deep	Superficial
Histology	Extensive abscess; hemorrhage/necrosis; no sinus tract formation	Three-layer granuloma; no hemorrhage/necrosis; sinus tract formation
Associated disease	Yes	No
Pathergy	Yes	Yes
Treatment	Systemic corticosteroids	Local anti-inflammatory, antibacterial agents
Course	Acute	Chronic

## References

[1] Wilson-Jones E, Winkelmann R (1988). Superficial granulomatous pyoderma: a localized vegetative form of pyoderma gangrenosum. J Am Acad Dermatol.

[2] Quimby S, Gibson L, Winkelmann R (1989). Superficial granulomatous pyoderma: clinicopathologic spectrum. Mayo Clin Proc.

[3] Akhras V, Sarkany R, Walsh S, Hyde N, Marsden R (2008). Superficial granulomatous pyoderma treated preoperatively with infliximab. Clin Exp Dermatol.

[4] Bennett M, Jackson J, Jorizzo J, Fleischer A, White W, Callen J (2000). Pyoderma gangrenosum: a comparison of typical and atypical forms with an emphasis on time to remission. Case review of 86 patients from 2 institutions. Medicine.

[5] Dobson C, Parslew R, Evans S (2003). Superficial granulomatous pyoderma treated with intravenous immunoglobulin. J Am Acad Dermatol.

[6] Hardwick N, Cerio R (1993). Superficial granulomatous pyoderma: a report of two cases. Br J Dermatol.

[7] Lachapelle J, Marot L, Jablonska S (2001). Superficial granulomatous pyoderma gangrenosum of the face, successfully treated by ciclosporine: a long-term follow-up. Dermatol.

[8] Lichter M, Welykj S, Gradini R, Solomon L (1991). Superficial granulomatous pyoderma. Int J Dermatol.

[9] Tollefson M, Cook-Norris R, Theos A, Davis D (2010). Superficial granulomatous pyoderma: a case in an 11-year-old girl and review of the literature. Ped Dermatol.

[10] Thami G, Kaur S, Punia R, Kanwart A (2002). Superficial granulomatous pyoderma: an idiopathic granulomatous cutaneous ulceration. J Eur Acad Dermatol Venereol.

